# Ten simple rules for creating and sustaining antiracist graduate programs

**DOI:** 10.1371/journal.pcbi.1010516

**Published:** 2022-10-13

**Authors:** Edgar Perez-Lopez, Larisa Gavrilova, Janice Disla, Melissa Goodlad, Dalena Ngo, Arabi Seshappan, Farhana Sharmin, Jesus Cisneros, Christopher T. Kello, Asmeret Asefaw Berhe

**Affiliations:** 1 Department of Mechanical Engineering, University of California, Merced, California, United States of America; 2 Department of Psychological Sciences, University of California, Merced, California, United States of America; 3 Department of Chemistry and Chemical Biology, University of California, Merced, California, United States of America; 4 Interdisciplinary Humanities Graduate Program, University of California, Merced, California, United States of America; 5 Department of Physics, University of California, Merced, California, United States of America; 6 Graduate Division, University of California, Merced, California, United States of America; 7 Department of Cognitive and Information Sciences, University of California, Merced, California, United States of America; 8 Department of Life & Environmental Sciences, University of California, Merced, California, United States of America; Carnegie Mellon University, UNITED STATES

## Abstract

In 2020, the combination of police killings of unarmed Black people, including George Floyd, Breonna Taylor, and Ahmaud Arbery, and the Coronavirus Disease 2019 (COVID-19) pandemic brought about public outrage over long-standing inequalities in society. The events of 2020 ignited global attention to systemic racism and racial inequalities, including the lack of diversity, equity, and inclusion in the academy and especially in science, technology, engineering, mathematics, and medicine (STEMM) fields. Racial and ethnic diversity in graduate programs in particular warrants special attention as graduate students of color report experiencing alarming rates of racism, discrimination, microaggressions, and other exclusionary behaviors. As part of the Graduate Dean’s Advisory Council on Diversity (GDACD) at the University of California Merced, the authors of this manuscript held a year-long discussion on these issues and ways to take meaningful action to address these persistent issues of injustices. We have outlined 10 rules to help graduate programs develop antiracist practices to promote racial and ethnic justice, equity, diversity, and inclusion (JEDI) in the academy. We focus on efforts to address systemic causes of the underrepresentation and attrition of students from minoritized communities. The 10 rules are developed to allow graduate groups to formulate and implement rules and policies to address root causes of underrepresentation of minoritized students in graduate education.

## Introduction

In 2020, Black Lives Matter protests across the world and disproportionately higher rates of Coronavirus Disease 2019 (COVID-19) infection and death among Black, Latinx, and Native American communities [1] once again brought to light systemic inequalities and the need to end systemic racism that exists in the broader community and in systems of higher education. Historically, western colleges and universities were created to serve mostly White people, and men, and have historically excluded Black, Indigenous, and people of color (BIPOC). Western institutions of higher education were created with, or at least benefited from, systems rooted in racism and White supremacy [2]. Although there have been efforts to combat racism and address systemic and structural problems associated with racism in higher education, evidence indicates that these efforts have not translated fully into increased representation of students of color in higher education [3,4]. Low enrollment rates of students of color in higher education continue to raise substantial concern among universities and colleges across the country [5].

Although the graduate student experience is stressful for almost all students, it carries an additional burden for students of color who face unique challenges and race-related barriers. Students of color experience racism, discrimination, microaggressions, and other exclusionary behaviors that are often perpetuated by well-intended peers, faculty, and supervisors and other university personnel [6,7]. These microaggressions generally include being treated like a criminal or a second-class citizen, having one’s intelligence underestimated, devaluation of one’s work, and feelings of isolation [8]. Many students of color report feeling unwelcome, invisible, and stigmatized which contributes to poor mental health and difficulties in completing their programs [9].

Academic institutions, in particular graduate programs, are in a unique position to address systemic racism. Indeed, racial diversity is considered to be one of the defining features of a university, particularly how graduate/undergraduate programs are ranked in terms of their curricula and how they address issues of equity and diversity [10]. The responsibility to advocate for minoritized students and fight racism, therefore, falls on all scholars.

**Fig 1:**
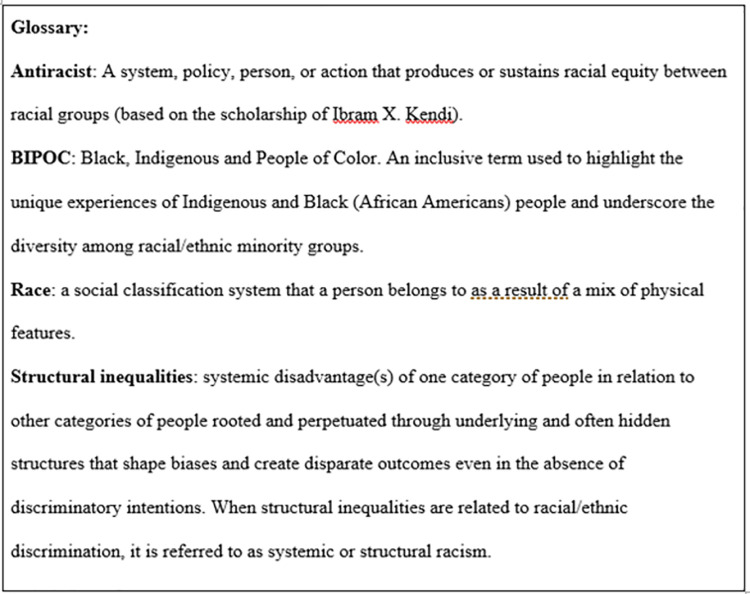
Glossary of terms encountered when discussing antiracism practices.

Following the Black Lives Matter protests around the world, we decided to work together to develop rules to help graduate programs develop antiracist practices in an effort to improve justice, equity, diversity, and inclusion (JEDI) in their graduate programs. This paper distills a year-long effort in achieving a more inclusive environment for graduate students from communities that are currently minoritized in the academy. We introduce 10 simple rules that are foundational in aiding graduate groups to implement antiracist practices within their own program or department. We present these rules as starting points for further conversation on best ways to eliminate root causes for systemic racism in higher education with the addition of common terms listed in Fig 1.

### Rule 1: Create an inclusive climate for recruiting and promoting the success of minoritized graduate students

Campus racial climate is described as an academic society’s current beliefs, judgements, and outlooks surrounding race, ethnicity, and diversity [3]. Campus racial climate can have an impact on the well-being and retention of minoritized graduate students [11,12]. When faced with issues of race, students often engage in self-censorship and question whether they belong within a program [12]. Evidence has shown that students participating in programs with pronounced emphasis on diversity and inclusion have higher retention rates than their counterparts [13,14]. Further, prospective applicants that connect strongly with their minoritized identity are more likely to apply to institutions that value a diverse climate [15,16]. Therefore, it is important to have an inclusive graduate community in order to recruit minoritized candidates with a clear and positive stance regarding racial diversity and equity. This stance is also important to promote minoritized student success from the moment they start their studies and throughout their graduate careers.

To improve the racial climate on campus, higher education institutions could mandate a race and ethnic studies course as part of the required course load for all registered undergraduate and graduate students. Previous studies examining the impact of curricular diversity initiatives on undergraduate students have found positive effects on students’ openness to cultural awareness, greater appreciation of multiple cultures, and interest in racial understanding [17]. In addition, higher education institutions could highlight multicultural and diverse perspectives for all faculty and staff, as done at our home institution. Instructors exposed to diverse perspectives have exhibited signs of personal growth as evidenced through attitudinal and curricular changes [18]. Students have benefitted from attending courses taught by faculty who have learned about multicultural course development. Programs may also include JEDI courses relevant to the field of study and opportunities for JEDI-related research, which can all contribute to creating a socially and academically supportive environment [19]. Students who benefitted from such courses report experiencing a greater sense of community, personal growth, and conflict resolution skills [14].

Finally, graduate programs should create inclusive environments that are sensitive to different cultural and language barriers. This can be accomplished through a variety of actions including offering online as well as in person English as a second language classes for those who are non-native speakers in order to address different language realities and creating spaces (e.g., virtual and in-person) for international student interaction and mentoring to promote integration and socialization processes.

### Rule 2: Intentionally recruit from diverse communities and remove barriers to entry for minoritized graduate students

While universities have made efforts to attract students of color, inequities and underrepresentation remain widespread, especially in STEM disciplines [3,4]. Results of more than 40 years of historic efforts to increase the number of BIPOC students recruited into the academy suggest that traditional means of student recruitment may be ineffective [20]. Students of color tend to face unique challenges in pursuing a higher education including cultural unease, viewing college campuses as hostile or indifferent, failure to succeed due to institutional bias, and feeling that pursuing an advanced degree will alienate them from their community [21,22]. However, direct recruitment of minoritized scholars may increase their rate of graduate school application and matriculation—as has been seen in biomedical graduate programs at both the University of Texas and Brown University [13,23].

Several strategies exist to address the unique challenges experienced by members of minoritized groups in higher education. First, universities can focus their efforts on outreach of available opportunities to diverse groups, including diversity preview weekends and developing relationships with local schools and colleges, minority serving institutions, and professional communities. Many states and regions have conferences and other meetings with special attention to minoritized scholars, and several national meetings are available as well, such as SACNAS, ABRCMS, and GEM. Many graduate programs and universities (including UC Merced) waive application fees for students recruited from meetings and organizations focused on championing JEDI.

It is important to remove any unnecessary barriers or biases that may be part of admissions processes, and there have been studies and discussions to identify and address systemic barriers and biases found in traditional admissions processes (see https://www.projectamiga.org/). For example, admissions criteria traditionally include standardized test scores to assess student knowledge and preparation [24]. However, standardized tests do not readily account for opportunity gaps and lack of access to resources for college preparation, career readiness, and citizenship [24]. Therefore, we should reconsider the current foundation of a decision-making system that reinforces extant power relations by applying criteria and processes that systematically favor the privileged. Indeed, graduate programs at UC Merced and around the country are reconsidering the costs versus benefits of standardized test scores, and many have stopped collecting GRE scores and instead moved to more holistic review processes that consider the unique constellation of factors and experiences that each applicant brings to the program and university.

### Rule 3: Establish empowering mentorship for graduate students throughout their graduate education and beyond

Mentoring is one of the most essential aspects of graduate education that can make or break graduate student success, which has impacts on faculty labs, graduate programs, and the university community as a whole. Effective mentoring provides psychosocial and career support, and it can mitigate the harmful effects of unsupportive culture and climate within a department, program, or the whole institution. Mentorship is a central factor in determining whether a minoritized student will apply for or complete graduate school [25–29].

Despite its importance, research indicates that mentorship can be problematic for minoritized graduate students [30]. Modern mentorship training programs like the Center for the Improvement of Mentored Experiences in Research (CIMER at https://cimerproject.org/cam-nrmn and practiced at UC Merced) suggest several best practices to make mentorship more inclusive and improve mentorship outcomes.

One of the most important practices is for students to be empowered to build teams of mentors, and thereby diversify their sources of support, their role models, and their academic and personal communities [31]. It is also important for universities to have resources and support systems for graduate students to establish and maintain healthy professional relationships, and to navigate conflicts with advisors, mentors, instructors, and other faculty in positions of power. Students from minoritized communities, especially women of color, have limited access to role models and mentors who look like them and who have similar personal and professional interests [32]. The adoption of team mentorship helps graduate students develop mentoring relationships with other individuals within and outside of their department, program, or institution.

Faculty often have limited numbers of mentors in their own academic lives, which may limit their experience with inclusive models of mentorship [33,34]. Mentorship training programs are designed to expose faculty to best practices culled from a wide range of experiences and mentorship contexts, such as individual development programs and techniques for maintaining clear and transparent communications and expectations. These programs are especially important for junior faculty beginning to advise students, formulate expectations for their advisees, and how best to convey and maintain expectations [35].

Programs should address mentorship of students from diverse backgrounds including racial minorities (ex. culturally aware mentoring training developed by CIMER). Inclusive mentorship can be recognized through faculty awards and disseminated by incentivizing trained and experienced mentors to teach other faculty and serve as role models.

### Rule 4: Establish and communicate clear expectations for student learning outcomes in graduate programs

Research indicates that there can be a lack of clarity and miscommunication in graduate programs when defining the specific steps and expectations for degree progress and completion [36], which can lead to misalignment in defining student success and unsatisfying advisor–advisee relationships [37]. The lack of clarity is not only with respect to formal assessments of student learning such as course material, qualifying exams, and scholarship requirements, but also the “hidden” curriculum that sets the norms of development as a member of the academic community. Clear guidelines for successful degree progress and completion are especially important for students with less prior experience in research universities and cultures. Delineating expectations clearly has shown to be an effective strategy at aiding scholarly growth at the College of Chemistry at University of California, Berkeley, where they found “no gap in chemistry” between publication rates of minoritized scholars and their counterparts [38].

Graduate programs may support academic progress and advancement for a diverse range of students [39] by establishing and communicating clear program expectations and guidelines. Policies and procedures should be specific, explicit, and clearly communicated, and the “hidden curriculum” should be addressed, e.g., how to find resources and solicit feedback from committee members and peers [40]. Faculty should have explicit discussions with students about expectations, advising styles, and how sociocultural backgrounds can influence expectations and experiences in graduate school. It is also important to monitor for implicit biases that may be embedded in the program curriculum and to create equitable and holistic evaluation criteria [41]. Strategies to alleviate or circumvent implicit bias would include explicit support (informal and formal) from faculty and administration to rethink curricular assumptions that may create inequities or pose barriers [42].

### Rule 5: Recognize and promote the diverse range of values and outcomes that graduate degrees have for students

Successful graduate degree outcomes are traditionally defined in terms of successful preparation for academic careers. However, success requires a more diverse range of outcomes for a more diverse range of students to more broadly reflect their cultural, racial, and socioeconomic backgrounds [43]. As one example, Latinx students often define success as surpassing the success of their parents and making contributions to their community, while Korean-American students define success as achieving the highest levels of academic achievement [44]. When minoritized students are placed in programs where definitions of success misalign with their own, they may choose to leave [45,46]; expanding the range of successful outcomes may lower attrition and increase graduation rates. Some scholars [47] argue that to achieve racial equity in higher education, the academic community must further develop equity-mindedness and positive race-consciousness. Race-neutral practices can unintentionally disadvantage racially minoritized students and perpetuate institutional racism.

Graduate advisors and faculty should appreciate the full range of possible career paths and values associated with their graduate program degrees. Graduate students, and especially minoritized graduate students, have been found to believe that their advisors prefer them to pursue academic research careers [48]. While advisors do prefer research careers in general, they were found to recognize and appreciate specific career paths for specific students. Graduate programs should instill a culture that explicitly values the range of graduate career paths that students may wish to pursue. University administration can help by hosting both academic and non-academic career conferences for graduate students, as done at UC Merced.

### Rule 6: Recognize and promote scholarship from students of diverse backgrounds

The importance of professional recognition in academia is self-evident and especially so for students in graduate programs seeking awards, honors, citations, and other accolades to help launch their careers. Some institutions and agencies have programs to recognize and promote minoritized scholars, and these programs have played important roles in broadening participation in academia. However, to further promote antiracism in graduate education, professional recognition can do more than just reward the scholar—it can also reward the scholarly value of diversity in research teams and activities. To help stop the perpetuation of systemic bias, JEDI values should not be isolated to programs and fellowships focused on minoritized graduate students. Instead, JEDI should be integrated into the solicitation and evaluation of all graduate reward programs, e.g., through the use of rubrics that make JEDI criteria explicit (as done at UC Merced) and intentional discussion of JEDI criteria during application and nomination reviews.

Graduate programs can support minoritized students by showcasing their innovations and contributions while highlighting the role of diversity in promoting innovative studies and research. Graduate programs should ensure that research related to minoritized scholarship is valued and cited [49]. Additionally, the critical role of diversity in fostering innovation can be promoted in the review of awards, fellowships, and other professional accolades. Advisors, letter writers, and departments can promote JEDI accomplishments and highlight their important contributions to scholarship. Funders can encourage diversity on research teams and prompt investigators to report on the value of diverse teams in producing research outcomes, especially for interdisciplinary teams and research that benefits from divergent thinking [50].

### Rule 7: Support communities of minoritized graduate students through coalition building and community engagement

Diverse coalitions have the ability to bridge societal divides [51–55] while promoting inclusivity. Students of color at predominantly White schools report unwelcoming environments, feelings of alienation, and a multitude of barriers to degree completion [56–59]. The support of coalition building is crucial because coalitions can promote the diversity of cultural experiences and values. Coalition building can provide marginalized minorities with a platform to be heard and a way to address marginalization and oppression in the community without fear of retaliation. In fact, an increase in minoritized student retention rates at one biomedical program at the University of Texas were partly attributed to the existence of such student support groups [13].

One approach to community building and engagement is to establish interest groups, social events, and community forums that provide opportunities for JEDI discussions to occur [60,61]. For example, JEDI reading groups or book clubs can provide opportunities to network and form grassroots movements in an academic setting. Another approach is to include representation from minoritized graduate students on academic and student affairs committees and to establish diversity committees to address racial incidents and evaluate the effectiveness of antiracism actions. Graduate students may be compensated for significant service commitments to account for the special and additional role that they play. Finally, graduate student housing is an important resource for communities of minoritized scholars to promote a sense of belonging, and counseling and psychological services can provide support that is tailored to the needs of minoritized students.

### Rule 8: Support minoritized graduate students in upholding their right to organize and call for action

Student activism has historically played an important role in improving conditions of minoritized scholars and in transforming academic institutions for the better. Most recently, student activism sparked by societal issues has contributed to attention to movements such as Black Lives Matter and the Me Too in the academy to shine a bright light on prevalence of racial discrimination and sexual harassment in society as well as academia. Student activism has also provided effective responses to issues such as racist fraternity parties and vandalism [62]. Supporting student activism as a high-impact practice can improve student retention, especially among first generation and minoritized students [63].

Universities can support student voices by explicating and upholding values of academic freedom. Units devoted to JEDI values can organize events and discussions including external speakers about academic freedom, freedom of speech, and the pursuant responsibilities to principles of community and mutual respect, as recently done at UC Merced. Guidelines can be provided on protocols for peaceful demonstration and protest, and campus leaders can work with faculty to advocate for student groups and help them to realize their goals.

### Rule 9: Amplify and address the voices of minoritized graduate students

Anecdotally, authors of this paper have noted concerns that words of minoritized scholars are often discounted or cast aside (many times by accident); this is detrimental to progress of research, as studies of teamwork have shown that diverse teams perform better than homogenous ones [64]. The perspectives and experiences that minoritized scholars bring to the academy are likely to be different than those of the majority [65], and they might pose challenges to existing power structures within our academic institutions. Uprooting systemic bias can be difficult work that puts demands on already strained limits to time and resources. It may be easier for administrators to hold off on addressing the challenges raised by minoritized graduate students, possibly with the hope that issues will resolve themselves over time.

An antiracist graduate program should listen to the needs and concerns of minoritized students who have little power or privilege in the hierarchical system of the academy. Faculty and program leadership should make conscious efforts to avoid hijacking or discounting concerns and discussions of race with anecdotes from other types of discrimination (e.g., gender, class) unless they are without employing intersectionality. Climate surveys can be administered regularly and consistently (as is done for all UC campuses), and program faculty and leadership should have regular meetings to discuss feedback and develop action plans to foster more inclusive and supportive environments [66]. Those plans should be transparent so graduate students are aware of actions being taken to address their concerns, and they are empowered to be a part of the process.

### Rule 10: Demand diverse leadership, representation, and accountability in graduate education

Leadership is essential to creating an inclusive workplace for minoritized students [67]. Because graduate program leadership is what can create the changes delineated in this paper, we believe it is essential that leadership reflect the changes needed to be implemented. JEDI values should be reflected in the leaders who are responsible for fostering an inclusive graduate community. Diversity is also valuable in broadening the range of leadership skills and perspectives [68] that can lead to a more productive, successful, and innovative academic enterprise. Diverse leaders can be role models for minoritized graduate students in that they provide example career paths and inspiration for aspiring to leadership roles. Diverse leadership can also improve the organizational culture and contribute to the overall success of the institution [67].

Cultivating diverse and accountable leadership in graduate programs is directly related to the diversity of the faculty. Programs should invest in leadership development to recruit diverse faculty into leadership positions [69]. Effective leadership of graduate programs also requires the development and dissemination of shared goals, priorities, and values with all members of the program and ensures that all members are heard. JEDI goals should be quantified and measured in strategic plans (as in the UC Merced campuswide strategic plan; https://strategicplan.ucmerced.edu), and criteria for evaluating leaders should include their effectiveness in achieving JEDI goals and addressing student concerns about harassment, bullying, and discrimination [11,70].

## Conclusion

Addressing racism in society is not easy. However, as graduate programs are the hubs where we cultivate the leaders of tomorrow, we have a responsibility to confront the ongoing reality of oppression and take action against exclusionary and toxic workplace behaviors, climates, and cultures. Graduate programs are positioned to be leaders in the progress for racial equity in the academy and beyond. To improve diversity in the graduate student pipeline, graduate programs must act strategically when addressing factors that contribute to underrepresentation of students from minoritized groups in graduate programs. It is our hope that the 10 rules outlined in this paper will motivate specific actions to confront racism and promote racial and ethnic belonging, access, and JEDI values in graduate programs.
